# Epidemiologic profile of measles in Central African Republic: A nine year survey, 2007-2015

**DOI:** 10.1371/journal.pone.0213735

**Published:** 2019-03-20

**Authors:** Alain Farra, Tuspin Nicephore Loumandet, Marilou Pagonendji, Alexandre Manirakiza, Casimir Manengu, Raphaël Mbaïlao, Severin Ndjapou, Alain Lefaou, Ionela Gouandjika-Vasilache

**Affiliations:** 1 National Mycobacteriology Laboratory, Institut Pasteur of Bangui, Bangui, Central African Republic; 2 Enteric Virus and Measles Laboratory, Institut Pasteur of Bangui, Bangui, Central African Republic; 3 Health Sciences Faculty, University of Bangui, Bangui, Central African Republic; 4 Epidemiology Unit, Institut Pasteur of Bangui, Bangui, Central African Republic; 5 World Health Organization Focal Point for Immunization, Vaccines and Emergencies, Bangui, Central African Republic; 6 Expanded Programme on Immunization, Ministry of Health, Bangui, Central African Republic; 7 Epidemiological Surveillance Service, Ministry of Health, Bangui, Central African Republic; 8 Pharmacy Faculty, Nancy, France; The University of Warwick, UNITED KINGDOM

## Abstract

**Introduction:**

Measles remains a major public health problem in many developing countries in which vaccination coverage is poor, as is the case in the Central African Republic (CAR). At the beginning of the 2000s, a surveillance system was established in the country, and samples from suspected cases are regularly tested in the laboratory for serological confirmation. Since 2007, when case-by-case monitoring with standardized laboratory databases and monitoring, was set up, no assessment have been performed. Therfore, 9 years later it seemed appropriate to make a first assessment. The aim of the study reported here was to describe the epidemiology of measles in the CAR on the basis of surveillance and laboratory data.

**Method:**

A descriptive retrospective study was conducted, based on the databases of the measles surveillance programme and of the Institut Pasteur laboratory in Bangui during the period 2007–2015.

**Results:**

During this study period, the surveillance programme notified 3767 cases. Of these, 2795 (75%) were sent for laboratory confirmation, and 24.6% (687/2795) were confirmed serologically. Of the 1797 cases of measles declared during this period by the surveillance programme, 1110 (61.8%) were confirmed clinically or by epidemiological linkage. The majority of confirmed cases (83.7%; 575/687) occurred in children under 10 years, over half of whom (44.2%; 304/687) were aged 1–4 years. Epidemics occurred regularly between 2011 and 2015, with > 10% of laboratory-confirmed cases. The rate of laboratory investigation was < 80% between 2011 and 2013 but nearly 100% in the other years.

**Conclusion:**

Measles remains a common, endemic illness in the CAR. Improved detection will require better measles surveillance, increased vaccination coverage, revision of the investigation forms to include the WHO case definition and training of the health personnel involved in case-finding in the field.

## Introduction

Measles is a highly contagious eruptive viral diseases that occurs only in humans. In 1980, before vaccination became widespread, there were estimated to be 2.6 million deaths due to measles per year [1]. The discovery towards the end of the 1960s of an anti-measles vaccine and its widespread use reduced the morbidity and mortality from this disease considerably. Between 2000 and 2014, 17.1 million deaths were avoided globally, making this vaccine the best public health investment to date [1]. According to the World Health Organization (WHO) Regional Office for Africa, the number of deaths from measles was also considerably reduced in the African Region, from a mean of one million cases per year between 1980 and 1989 to fewer than 100 000 cases per year between 2006 and 2009 [2]. This was due to improved vaccination coverage, from 56% to 69%, an increase from 4 to 13 in the number of countries with vaccine coverage > 90% and a decrease from 8 to 2 in the number of countries with vaccine coverage < 50% [2].

Nevertheless, measles remains an important cause of death among young children in Africa, even though there is a reliable, effective, inexpensive vaccine [3]. The fourth Millennium Development Goal is to eradicate measles by 2020 in all regions of the world, with a strategy based on strengthened vaccination services, introduction of a booster dose, strengthening of surveillance systems by laboratory confirmation of suspected cases and improving the care of patients [2–4]. This WHO-recommended strategy was adopted by the Central African Republic (CAR) and implemented in the programme of integrated disease surveillance and response. Since 1979, one dose of the vaccine against measles has been administered free of charge to infants < 9 months in the Expanded Programme on Immunization introduced in the CAR by WHO and UNICEF. The mean coverage with the vaccine between 2007 and 2015 was, however, only 49% (25–59%), and many suspected cases of measles are reported throughout the country each year by the surveillance programme [5]. Since 2007, when case-by-case monitoring with standardized laboratory databases and monitoring, was set up, no assessment have been performed. Therfore, 9 years later it seemed appropriate to make a first assessment.

The aim of this study was to characterize the epidemiology of measles in the CAR on the basis of surveillance data and results from the laboratory for measles serology at the Institut Pasteur of Bangui.

## Materials and methods

### Study description and population

A retrospective descriptive study was conducted of data from the Enteric Virus and Measles Laboratory at the Institut Pasteur of Bangui and from the WHO epidemiological surveillance service in Bangui between 1 January 2007 and 31 December 2015. The measles surveillance system in the CAR covers the entire country, and surveillance in all health districts ensures collection of data on all cases of measles. The target population were cases that filled the WHO criteria for suspected measles, i.e. fever, maculopapular rash and one of the following signs: cough, coryza or conjunctivitis. Seven health districts in the country reported cases within 28 days of the beginning of a rash.

### Sampling and transport

From each patient, 5 mL of venous blood were taken by trained focal points and placed into two sterile tubes, labelled with the full name of the patient and the date. In certain districts, the blood sample was left for 2 h at ambient temperature, and the serum was collected and placed in two tubes labelled as above. If the transport was delyed, the samples were stored at 4–8°C in a frige available at the district or health centre level. The blood or serum samples were transported in refrigerated vaccine containers at 4–8°C by the focal points, who ensured transfer to the laboratory within 3 days of sampling. Urine samples and throat swabs, which are useful for diagnosis, were not taken regularly due to lack of proper equipment and because they are more difficult to obtain than blood samples.

### Laboratory investigations

When samples were received at the laboratory, tubes containing whole blood were centrifuged at 3000 × *g* for 10 min, and the serum was placed in two 1.5-mL cryo-tubes, as were serum samples, labelled with the date the sample was received and an identification number (e.g. CAR-RS7-COM-2014-212), indicating the country (CAR), the health district (e.g. RS7) and region of origin (e.g. COM), the year and the order of reception (e.g. 212). One cryo-tube was placed in an archive box according to its order of reception and stored in a freezer at– 20°C, and the other was tested directly or stored at 4°C if the analysis was delayed.

#### Serological tests

Measles-specific immunoglobulin M (IgM) was identified by enzyme-linked immunosorbent assay (Siemens Enzygost measles anti-measles virus/IgM kit, provided by WHO). According to the WHO diagnostic algorithm, if a test result is negative, a test is done for anti-rubella IgM; if the result for measles is indeterminate, the sample is tested again. If the second test results are negative or still indeterminate, rubella serology is done. If the result is positive for measles, rubella serology is not done. The Enteric Virus and Measles Laboratory at the Institut Pasteur of Bangui is a WHO national laboratory for measles since 2007 and participate early at the WHO proficiency testing programme, wich it never failed.

### Data collection and analysis

All results were entered onto forms and compiled in the laboratory database. A copy of the results was sent to WHO, the Ministry of Health and the health district that sent the sample. The results of the serological tests and sociodemographic characteristics of the patients were analysed with Epi info version 7, and Excel 2013 was used to design figures and tables.

### Variables definitions [2]

#### Suspected cases

All persons with a maculopapular generalized rash and fever, and one of the following signs: cough, coryza, conjunctivitis.

#### Declared cases

All cases recognized as measles by the surveillance service (laboratory-confirmed and cases declared without testing).

#### Laboratory confirmed cases

A laboratory-confirmed case is one in which measles-specific immunoglobulin M has been confirmed serologically in a person who has not been vaccinated within the previous 30 days.

#### Cases declared without testing

Proportion of declared cases of measles not tested in the laboratory but considered probable measles cases on the basis of clinical criteria or epidemiological links.

#### Samples received in good conditions

Sampling containing 5ml of blood or serum in a dry tube on which are inscribed the name, the patient's name, the date of the sample and arriving at the laboratory with a temperature between 4–8° C.

### Ethical considerations

The surveillance programme was approved by an expert ethical committee of the Ministry of Health (Decree 0277/MSPP/CAB/DGSPP/DMPM/SMEE of 5 August 2002) [6]. Data were used anonymously, with strict respect for the confidentiality of the patients included.

## Results

### Sociodemographic and clinical characteristics of the study population

Between 2007 and 2015, 2795 suspected cases of measles were registered at the laboratory, of which 1445 (51.7%) were female and 1350 (48.3%) male, for a sex ratio of 0.93. The mean age was 6.3 years (range, 14 days to 66 years). Most samples were sent from health district 7 (1098/2795, 39.3%) and the fewest from health district 6 (123/2795, 4.4%). Skin eruptions were the most reported signs in 99.8% of cases (2773/2795) (Table 1).

**Table 1:** Demographic and clinical characteristics of the study population.

Characteristic	No.	%
Age group (years)		
< 1	289	10.3
1–4	973	34.8
5–9	981	35.1
10–14	363	13.0
15–19	71	2.6
> 20	118	4.2
Sex		
Male	1350	48.3
Female	1445	51.7
Health district		
1	393	14.1
2	330	11.8
3	330	11.8
4	341	12.2
5	180	6.4
6	123	4.4
7	1098	39.3
Clinical sign		
Cutaneous eruptions	2773	99.8

### Prevalence of measles in the samples received

Of the 2795 samples sent to the laboratory for analysis for anti-measles virus-specific IgM, 24.6% (687) were positive, 72.5% (2026) were negative, and 2.9% (82) were indeterminate. Although twice as many samples were sent during the last 2 years, with 733/2795 (26.2%) in 2014 and 749/2795 (26.7%) in 2015, the proportion of positive results did not increase. Overall, the rate of positive samples was low, at 24.6%, with a very high rate in 2013 (55.4%) and a very low rate in 2011 (5.8%). The rate was also low in 2015 (12.8%) but with a high proportion of indeterminate results (6.4%) (Table 2).

**Table 2:** Serological results for measles, CAR, 2007–2015.

Year	Suspected cases (No. (%))	Serological result (No. (%))		
		Positive Indeterminate Negative		
2007	268 (9.6)	11 (4.0)	7(2.6)	250 (93.3)
2008	111 (4)	10 (9.0)	5 (4.5)	96 (86.5)
2009	126 (4.5)	14 (11.1)	3 (2.4)	109 (86.5)
2010	95 (3.4)	2 (2.1)	0 (0.0)	93 (97.9)
2011	162 (5.8)	72 (44.4)	5 (3.1)	85 (52.5)
2012	190 (6.8)	67 (35.3)	3 (1.6)	120 (63.2)
2013	361 (13)	200 (55.4)	5 (1.4)	156 (43.2)
2014	733 (26.2)	215 (29.3)	6 (0.8)	512 (69.8)
2015	749 (26.7)	96 (12.8)	48 (6.4)	605 (80.9)
Total	2795 (100)	687 (24.6)	82 (2.9)	2026 (72.5)

### Overall results of measles surveillance

Countrywide measles surveillance showed that samples were taken from only 74% (2795/3767) of the notified cases, which was higher in 2007 (88.7%) but lower in 2011–2013 (21.2%, 55.9% and 60.5%). In 2014 and 2015, however, all cases of measles were declared after a positive laboratory result (Table 3).

**Table 3:** Results of measles surveillance, CAR, 2007–2015.

Year	Suspected cases notified No. (%)	Suspected cases sampled No. (%)	Declared cases No. (%)	Laboratory-confirmed cases No. (%)	Cases declared without testing No. (%)	Declared cases per 100 000 inhabitants
2007	302	268 (88.7)	49 (16.2)	11 (22.4)	38 (77.6)	1.17
2008	117	111 (94.9)	12 (10.2)	10 (83.3)	2 (16.7)	0.28
2009	126	126 (100)	14 (11.1)	14 (100)	0	0.25
2010	95	95 (100)	2 (2.1)	2 (100)	0	0.04
2011	762	162 (21.2)	679 (89)	72 (10)	607 (90)	15.12
2012	340	190 (55.9)	141 (41)	67 (47.5)	74 (52.5)	3.08
2013	596	361 (60.5)	596 (100)	200 (34)	396 (66)	12.77
2014	733	733 (100)	215 (29)	215 (100)	0	4.45
2015	749	749 (100)	96 (12.8)	96 (100)	0	1.95

### Epidemiological aspects of laboratory-confirmed cases, 2007–2015

#### Age and sex

**Fig 1:**
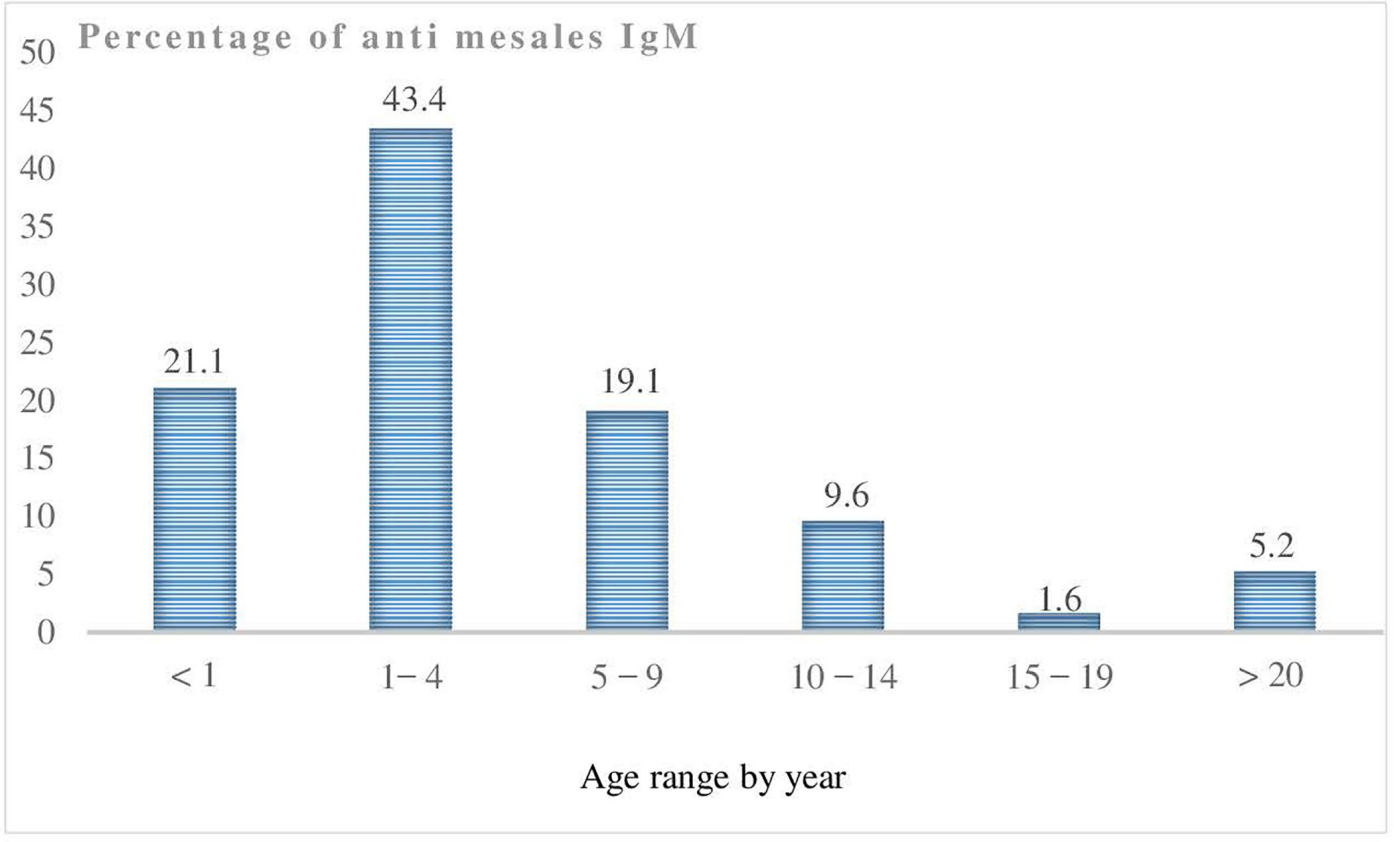
Distribution by age range of patients with anti-measles IgM, CAR, 2007–2015).

Anti-measles IgM was maximum in children aged 1–4 years (43.4%) but was also high in those under 1 year of age and in children aged 5–9 years. Low rates were seen in older children and adults (Fig 1).

Of the 687 laboratory-confirmed cases, 52.8% (363) were female and 48.2% (324) were male; however, the rate of positivity was similar: 25% (363/1445) of the tests for IgM among females and 24% (324/1350) among males were positive.

#### Vaccination coverage

**Fig 2:**
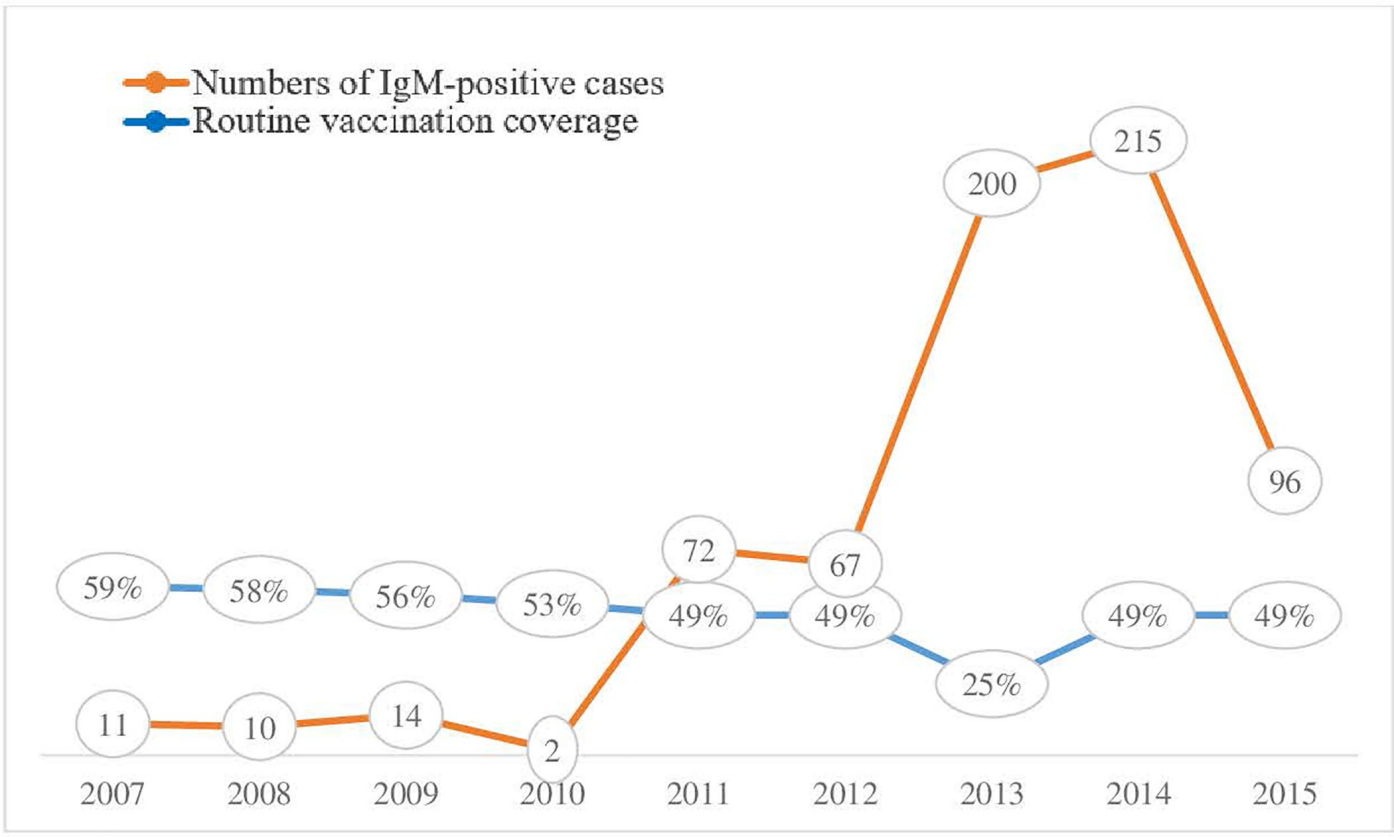
Numbers of IgM-positive cases and routine vaccination coverage (%), 2007–2015).

Before 2010, when vaccination coverage was > 50%, IgM was found in few cases, with a maximum of 14 cases in 2009. After 2011, when vaccination coverage dropped below 50%, detection of anti-measles IgM clearly increased (72 cases), with a large increase after 2013 (≥ 200 cases) when vaccination coverage was only 25% (Fig 2).

#### Seasonality and health districts

**Fig 3:**
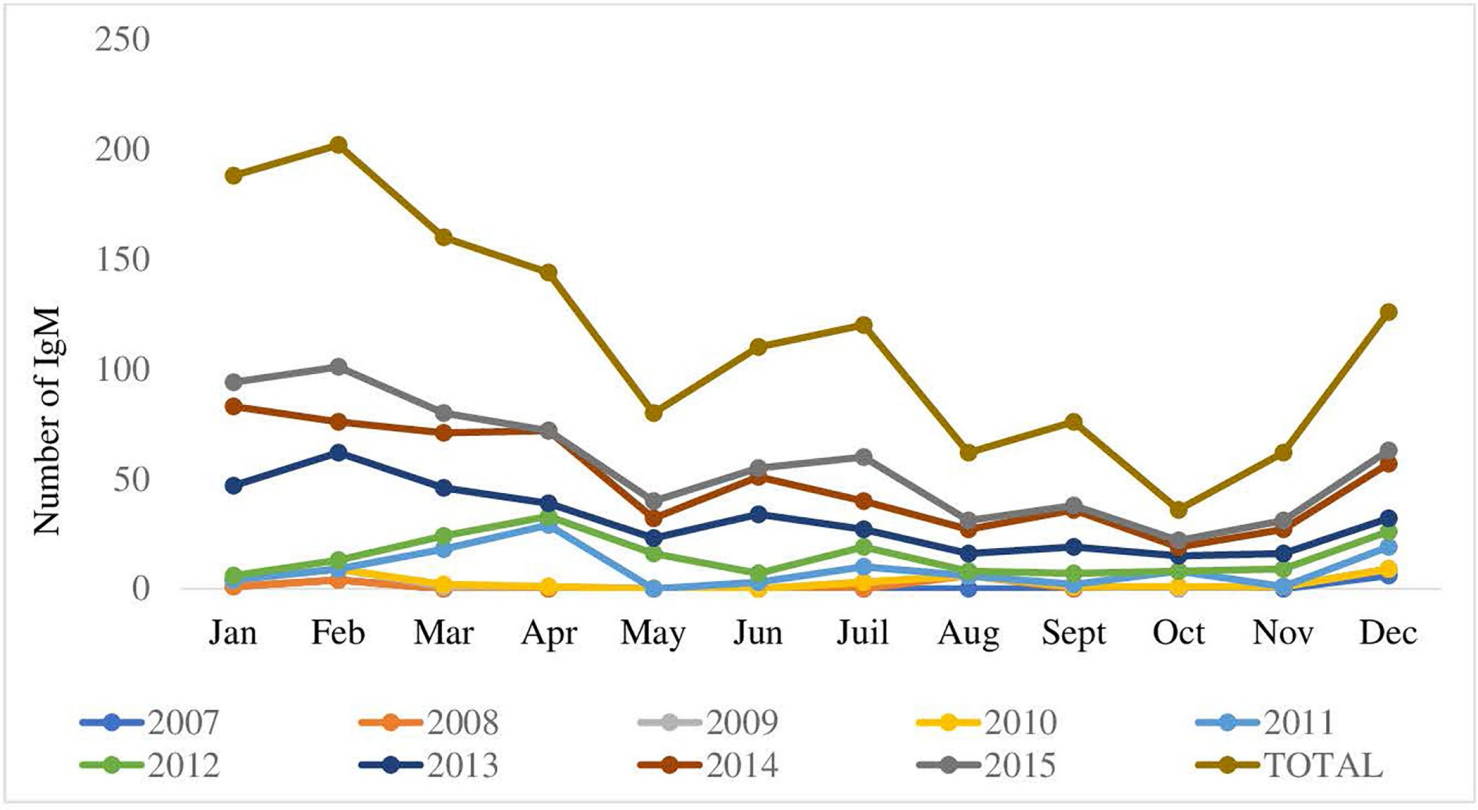
Distribution of anti-measles IgM per month, 2007–2015.

Cases of measles were found each year, but mostly between December and April and with a peak in June and July (Fig 3).

**Fig 4:**
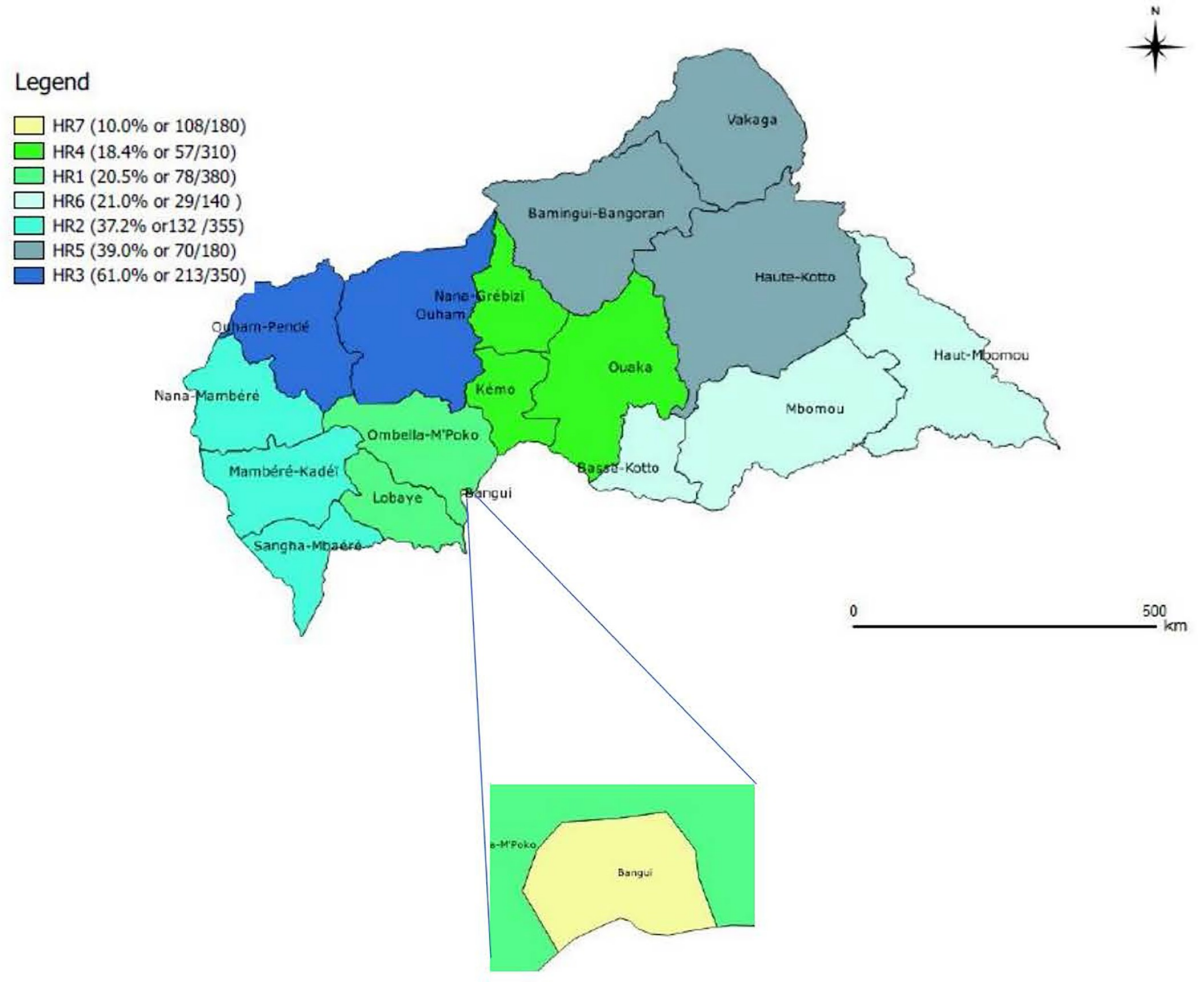
Distribution of confirmed measles cases by health region, 2007–2015).

The highest rate of IgM positivity was found in health district 3 (61%), followed by districts 5 (39%) and 2 (37.2%). Although the most suspected cases were found in district 7, the rate of IgM positivity was the lowest (10%) (Fig 4).

#### Performance indicators for surveillance of measles

Laboratory results were available within the delay recommended by WHO (≥ 80% before 7 days), except in 2013 and 2014. The samples were received in good condition but within the prescribed delay only in 2007 (Table 4).

**Table 4:** Main performance indicators for laboratory surveillance, 2007–2015.

Year	No. of samples	Transport ≤ 3 days (≥ 80%)	Samples receive in good condition (≥ 80%)	Results available within 7 days (≥ 80%)
2007	268	267 (99.6)	261 (97.4)	215 (80.5)
2008	111	67 (60.4)	109 (98.2)	110 (99.1)
2009	126	53 (42.1)	118 (93.7)	124 (98.4)
2010	95	65 (68.4)	95 (100)	89 (93.7)
2011	162	116 (71.6)	162 (100)	157 (98.1)
2012	190	117 (61.6)	190 (100)	183 (96.3)
2013	361	250 (69.3)	361 (100)	278 (77.0)
2014	733	494 (67.3)	731 (99.9)	523 (74.7)
2015	749	547 (73)	748 (100)	682 (91.1)
Total	2795	1976 (70.6)	2775 (99.2)	2361 (84.4)

The surveillance data showed that, in 2007 and 2014, districts had been able to send at least two samples to the laboratory, while less than 80% of districts had been able to do so in the other years (Table 5). After 2011, the proportion of samples that tested positive was regularly > 10%, indicating an epidemic of measles in the country, although the situation appeared to have improved in 2015. The rate of investigation per 100 000 inhabitants was ≥ 2 throughout the study period. Between 2011 and 2013, samples for diagnosis were taken from < 80% of suspected cases, while the proportion was nearly 100% in the other years (Table 5).

**Table 5:** Performance indicators for the measles surveillance system, CAR, 2007–2015.

Year	Districts that sent ≥ 2 samples (%) (≥ 80%)	Rate of investigation per 100 000 inhabitants	Rate of laboratory investigation (≥ 80%)	Laboratory-confirmed cases (≤ 10%)
2007	83.3	6.4	88.7	4
2008	53.3	2.6	94.9	9
2009	63.3	2.9	100	11.1
2010	70	2.1	100	2.1
2011	56.6	3.6	21.2	44.4
2012	76.6	4.1	55.9	35.3
2013	60	7.7	66.5	55.4
2014	83.3	15.4	100	29.3
2015	76.6	15.4	100	12.8
Mean	68.8	6.6	80.8	22.6

## Discussion

This study was possible thanks to the measles surveillance system set up in CAR which covers the entire territory. Surveillance in health regions has made it possible to collect all suspected cases of measles, as defined by WHO. Although clinical information was limited to the presence of rash, in the laboratory, the techniques were used in accordance with WHO recommendations and the results obtained can be considered reliable because they meet the requirements of the WHO performance indicators and are regularly evaluated.

The majority of reported cases were aged less than 10 years old: 35.1% between 5 and 9 years old and 34.8% between 1 and 4 years old. The results reported in most African countries have confirmed a clear predominance of cases below 10 years, which shows that the highest suspicion of cases is reported in childhood [7, 8]. The sex ratio was close to 1 for all suspected cases (1350/1445; 0.93) as well as in confirmed cases (324/363; 0.89). Others studies conducted in Africa corroborate our results [8, 9].

The majority of reported cases were notified in the HR7, probably because of the geographic proximity with Institut Pasteur of Bangui, which would facilitate the transfer of the samples. The only clinical sign that was reported was rash, in 99.2% of cases. However, others surveillance system in different countries include more relevant signs such as cough, fever, runny nose and conjunctivitis in their case report forms [9–10].

Only 24% (687/2795) of cases were confirmed in the laboratory. This prevalence is similar to that found in Senegal between 2004 and 2013, which was 21.4%, and falls between those in Nigeria (16%) and Ethiopia (36.7%) [8, 9, 11]. These low proportions indicate that the signs recommended by WHO for identifying cases are nonspecific for measles, as other types of eruptive fever, such as rubella, roseola and scarlet fever, have the similar symptoms [12].

Anti-measles IgM was found throughout the year but predominantly from December to April, with a peak in February, during the dry season in the CAR. Similar observations were made in Burkina Faso, with a peak in March [13], and in Italy, with a peak in April [14].

Health district 3 appeared to be the worst-affected region, with 61% of cases positive for anti-measles IgM, and a study should be carried out to identify the determinants of this high prevalence. Our finding that most cases were reported from health district 7 corresponds to its population density, which includes the capital, Bangui, and its suburbs, and is also due to its proximity to the Institut Pasteur, which facilitates transport of samples.

Vaccination is the main strategy for protecting populations against measles; however, vaccination coverage in the CAR (49%) is insufficient. Furthermore, the single dose given at the age of 9 months within the Expanded Programme on Immunization does not result in seroconversion in 82–95% of vaccinated infants [2, 15]. Of the 687 patients in our study who were found to have anti-measles IgM, 117 (25.8%) had received at least one dose of the vaccine. In studies in the south of France [16] and in Senegal [17], 15% and 11.5%, respectively, had received a dose of vaccine. These findings indicate that one dose does not ensure satisfactory protection and protects only part of the vaccinated population. Two factors may be involved: an inadequate response to the vaccine and the difficulty of maintaining the cold chain in many countries of Africa.

During the 9 years of the study, 3767 suspected cases of measles were reported, corresponding to a mean of 418 cases per year, with wide variation from one year to another. In south-west Nigeria, 10 187 suspected cases were reported in 2007, for a mean of 1697 cases per year [9]. The difference from our results may be demographic, as Nigeria is the most populous country in Africa, with 186 053 386 inhabitants in 2016, whereas the CAR is one of the least populous countries on the continent, with < 5 000 000 inhabitants and sparse population density outside of Bangui [18]. Furthermore, the health systems may differ. In the CAR, there are few systems for notification, such as detection of suspected cases in families by local authorities, and only cases seen at consultations are notified. Patients usually present only for severe health problems, and eruptive fever, particularly if it is nonspecific, is often treated in the family. Of the 3767 suspected cases, 1797 (48%) were confirmed by either biological tests or by clinical or epidemiological criteria when a laboratory diagnosis was not available. The mean prevalence was 4.38 cases per 100 000 inhabitants per year. This is much lower than that in Nigeria (8.81 cases) between 2007 and 2012 [9] but similar to that between 2003 and 2013 in South-East Asia (5.89 cases) [19]. The results for the CAR therefore appear to be intermediate, although the prevalence is possibly underestimated and might be close to that in Nigeria.

The delay in sending samples to the laboratory was satisfactory only in 2007, when 100% of samples were sent within the prescribed time. The target of 80% required by WHO was not reached in the other years, with a very low rate of 42.1% in 2009. These results are associated with poor roads, long distances and inadequate public transport in rural areas, as the focal points do not have private means of transport and use buses to transport samples. In certain areas, there is no public transport for several days, due not only to the poor roads but also sometimes to lack of security. Once a sample has been taken, therefore, it often cannot be transported immediately. A survey should be conducted of the situation in the districts that send few samples per population size and send less than the threshold of two samples.

The threshold of 10% IgM positives, above which an epidemic is declared, was exceeded throughout the study, except in 2007 (4%), 2008 (9%) and 2010 (2.1%). The CAR is thus still undergoing epidemics of measles, as in Nigeria [9] and France [16] since 2008 and also in most other regions of the world [20, 21]. The recommended investigation rate of ≥ 2 per 100 000 inhabitants was reached throughout the study, but the 80% rate of laboratory investigation was not attained in 2011–2013, when cases were declared only on the basis of clinical and epidemiological criteria. This may be associated with the sociopolitical problems of that period, which hampered systematic transport of samples to the laboratory. In the Philippines, the establishment of sentinel sites for notifying all suspected cases, the national surveillance policy and laboratory identification of cases increased the proportion of laboratory-confirmed cases from 29.5% to 88.6% [21].

## Conclusion

Measles remains a common, endemic illness in the CAR because of inadequate vaccination coverage. In this study, only 24.6% of reported cases were confirmed in the laboratory. Children under 10 years were most affected, with no difference between the sexes. The disease is seen most often during the dry season, with a peak in February, and most confirmed cases were reported from health district 3, which includes Bangui and its suburbs. Better control of measles in the CAR will require increased vaccination coverage, revision of the investigation forms to include the current WHO case definition and training of health personnel involved in case-finding in the field.

## Supporting information

S1 Dataset Measles anonymized laboratory data base. (XLSX)

S2 Dataset Measles EPI data base 2007–2012. (XLSX)

S3 Dataset Measles EPI data base 2013–2015. (XLSX)
